# Using triallelic SNPs for determining parentage in North American yak ( *Bos grunniens*) and estimating cattle ( *B. taurus*) introgression

**DOI:** 10.12688/f1000research.25803.2

**Published:** 2020-10-29

**Authors:** Ted Kalbfleisch, Jessica L. Petersen, R. G. Tait Jr., Jiansheng Qiu, Veronica Basnayake, Peter H. Hackett, Michael P. Heaton

**Affiliations:** 1 Department of Veterinary Science, University of Kentucky, Lexington, Kentucky, 40546, USA; 2 Department of Animal Science, University of Nebraska-Lincoln, Lincoln, Nebraska, 68583, USA; 3 Neogen Genomics, Lincoln, Nebraska, 68504, USA; 4 USYAKS Association, Livermore, Colorado, 80536, USA; 5 U.S. Meat Animal Research Center, Clay Center, Nebraska, 68933, USA

**Keywords:** Yak, Introgression, Parentage Testing, SNP test

## Abstract

**Background:** Genetic testing for pedigree accuracy is critical for managing genetic diversity in North American (NA) yak ( *Bos grunniens*), a population expanded mostly from imported zoological park specimens.  DNA testing also enhances species conservation by identifying recent *B. taurus* F1 hybrid ancestors (within three generations).  Biallelic single nucleotide polymorphisms (SNPs) can accomplish either task, but increases the marker count and costs necessary to achieve both.  Our aim was to identify novel, multifunctional, triallelic yak SNPs (tySNPs), with each having two alleles for yak parentage testing, and a third allele for identifying recent cattle introgression.

**Methods:**  Genome sequences were aligned to the cattle UMD3.1 assembly and SNPs were screened for 1) heterozygosity in a NA and a Chinese yak, 2) a third allele at high frequency in cattle, and 3) flanking sequences conserved in both species.  Subsequently, tySNPs were filtered for unique alignment to the haplotype-resolved F1 yak assembly.  Allele frequencies were estimated in a subset of 87 tySNPs by genotyping 170 NA yak.

**Results:**  We identified 610 autosomal tySNPs, distributed in 441 clusters with 5 Mb average genome spacing.  The average NA yak minor allele frequency was high (0.296), while average introgressed cattle alleles were low (0.004).  In simulations with tySNPs, 28 were sufficient for globally-unique animal identification (P _I_=5.81x10 ^-12^), 87 were able to exclude 19 random bulls from parentage at the 99% level without using the dam’s genotype (P _E_=5.3x10 ^-4^), and 87 were able to detect F1 hybridization events after three generations of yak backcrosses (1/16th *B. taurus* germplasm).

**Conclusions**:  Identifying animals, determining parentage and detecting recent hybridization events was efficient with as few as 87 tySNPs.  A similar triallelic approach could be used with other bottlenecked *Bos* species that hybridize with cattle, such as NA plains bison ( *B. bison*).

## Introduction

Domestic yaks ( *Bos grunniens*) are native to the Qinghai–Tibet Plateau of Central Asia and valued around the world for their meat, fiber, milk, fuel, wool, transportation, predator protection, and as pets ^1^. The global yak population is large and diverse with upwards of 14 million domestic yak, and 15,000 wild yak ( *Bos mutus*) ^2^. In contrast, the North American (NA) yak population is small and narrow with only 2,000 to 5,000 yak, all of which are domestic. This herd has mostly arisen from a few dozen animals imported from public and private European zoological parks to NA zoos around the turn of the 20th century. The lack of source diversity may have also compounded the problem. For example, the Smithsonian National Zoo (SNZ) in Washington, DC imported their first yaks in 1898 from the Zoological Society of London, England ^3^. For 23 years the closed SNZ herd was expanded by breeding and surplus animals were sent away until the zoo received a single new yak breeding bull from Parks Canada. This Canadian bull descended from yaks kept at Woburn Abbey in England, approximately 50 miles from the original London Zoo source ^4^. Other than zoological park sources, additional yak germplasm has not been introduced to the NA herds due to strict federal regulation barriers placed on the importation of live animals, embryos, and semen. Thus, highly related surplus yaks from limited introductions are the apparent founders of the current NA yak population. This narrow genetic base of the NA yak population is an ongoing concern for maintaining genetic diversity to protect the health and vigor of these herds. 

The challenge of maintaining genetic diversity within NA yaks is increased by occasional hybridization with cattle ( *Bos taurus*). The issues arising from yak and cattle hybridization predates their export from the Qinghai–Tibet Plateau. The production of yak and cattle hybrids has been practiced for 3000 years, starting with the Yin Dynasty ^5, 6^. These ancient hybridization events account for the 2–3% *Bos taurus* alleles present in domestic yak today ^7^. 

Genetic management of ancient *B. taurus* introgression in domestic yak is not typically a concern for breeders. However, in the small NA yak population there are also documented cattle introgression events in the early 20th century ^8^, as well as undocumented introgression from occasional producer-directed efforts to introduce cattle traits like coat color and carcass yield. In some instances, extant animals may be only a couple of generations removed from a previous F1 cattle-yak hybridization event. Thus, identifying and documenting these recently hybridized animals (within three generations) is essential for preserving authentic *Bos grunniens* germplasm while producing healthy, genetically diverse animals.

SNP-based tools provide new opportunities for more precise genetic management of a given livestock species. Advances in SNP discovery and testing have made it routine to verify parentage, identify animals, and traceback diseases in cattle, sheep, horse, and swine ^9– 12^. Previously, yak parentage testing had used *Bos taurus* derived microsatellite markers (i.e., short tandem nucleotide repeats) ^13, 14^. However, publicly available whole genome sequence (WGS) data from Chinese and NA yak, together with a haplotype-resolved F1 yak-cattle assembly, made it possible to identify informative NA yak SNPs *in silico* for the potential development of parentage SNP markers ^15– 17^. The ultimate utility of a set of parentage SNPs may be measured by their success in accomplishing the most challenging scenario: “one-parent traceback.” For example, using DNA from a lamb carcass to identify its true sire in a multi-sire mating system, but not having the dam’s DNA available to perform the trio’s analysis ^9^. Identifying the offspring’s sire requires the genetic exclusion of all other males exposed to the dams. This approach is based on the principle that the true sire must share an allele with the offspring at every site tested ^18^. Thus, when an offspring and a potential sire are homozygous for different alleles at the same site, the potential sire is excluded from parentage. The ideal SNP set for one-parent parentage testing has markers with a minor allele frequency (MAF) greater than 0.30, is evenly spaced across the genome, and has SNPs with highly conserved flanking DNA sequences for efficient and accurate genotyping ^9^. If available, such yak biallelic parentage SNPs would allow producers to access commercial high-throughput SNP genotyping, use multiple-sire pasture breeding strategies, verify pedigrees, and establish unique animal identification information that could be used for tracing if needed.

**Figure 1.**
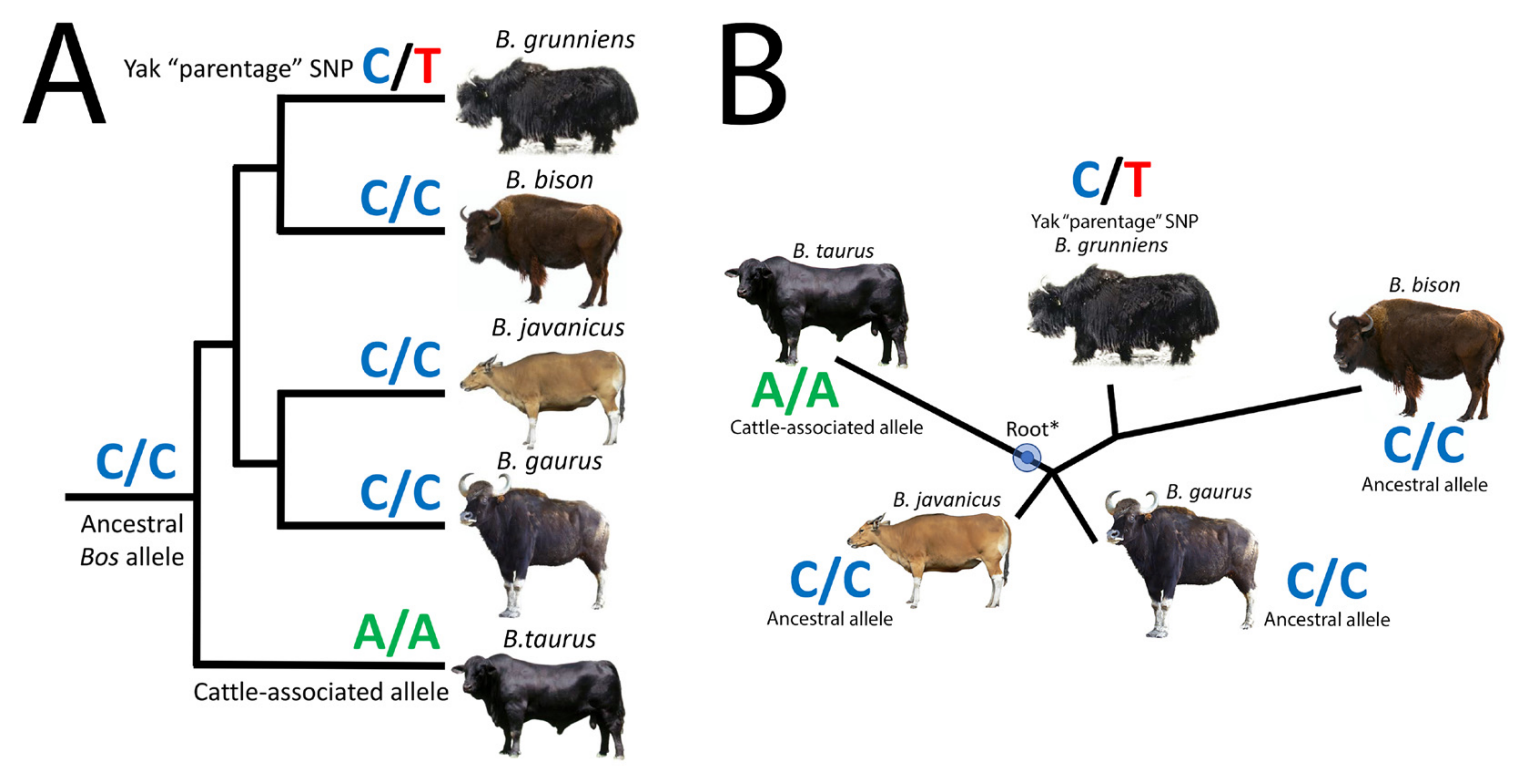
Hypothetical genotype distributions of a specialized triallelic SNP for determining yak parentage and estimating *B. taurus* introgression. *Bos* species trees are based on those published by Wu *et al*. (Wu *et al*., 2018. Nat Ecol Evol 2, 1139–45). ( **A**) Tree based on whole autosome sequences with the randomized axelerated maximum likelihood (RAxML) method. ( **B**) Tree based on whole autosome sequences with the accurate species tree algorithm (ASTRAL) method with a 100-Kb non-overlapping sliding window. *The tree is rooted at the base of the water buffalo ( *Bubalus bubalus*) branch. (Figure1_BosTrees.TIF)

Markers appropriate for biallelic yak parentage SNPs do not typically provide information about *B. taurus* introgression, which would require an additional set of biallelic SNPs thereby increasing cost while limiting choice of platforms suitable for lower throughput. However, based on our previous interspecies alignments of WGS ^19^, we hypothesized that some biallelic parentage SNP sites in yak may also align with a nearly monomorphic, alternative allele in *B. taurus*. In this hypothetical triallelic SNP system, the evolutionary source of all three alleles could be inferred by estimating frequencies in samples of *Bos* species, a genus that diverged 5 million years ago. This idea is consistent with the evolution of the *Bos* genus as a complex of genetically interconnected species with shared evolutionary trajectories ^20^. For example, an ancestral *Bos* allele would be common in most of the extant *Bos* species including yak ( Figure 1A, nucleotide “C”). A yak-associated allele used for parentage testing would have arisen since the time of the most recent common ancestor for *Bos* species ( Figure 1A, nucleotide “T”). Likewise, a different *B. taurus*-associated allele would have arisen in the same time frame ( Figure 1A, nucleotide “A”). The evolutionary distance between *B. taurus* and *B. grunniens* may be sufficient for each species to have evolved their own distinct alleles at the same genomic sites, allowing identification of novel triallelic yak parentage SNPs (tySNPs) ( Figure 1B). If a sufficient number of tySNPs (e.g., one in 26 million) could be identified with distributed genome spacing, high MAF in yak, and high specificity in *B. taurus*, it would be possible to develop a maximally-informative genetic test with a minimal set of about 100 SNPs. This only requires finding one high-quality tySNP per 26 million genomic positions and This would be ideal for a livestock species whose breeders do not currently have access to higher-density genome-wide SNPs and genotyping technologies.

Here we describe a panel of 610 tySNPs for use in animal identification, parentage testing, and for estimating recent *B. taurus* introgression events in NA yaks. Markers were sequentially filtered for having: 1) identical heterozygous genotypes in a NA and a Chinese reference yak, 2) identical homozygous genotypes in the other non-taurine *Bos* species, 3) a third high-frequency allele in *B. taurus*, 4) conserved flanking sequences in *Bos* species, and 5) a unique location in the *B. grunniens* genome. A representative trial set of 87 tySNPs were used to estimate performance in NA yak with a MALDI-TOF genotyping platform. Based on these estimates, we performed computer simulations to predict the power to determine parentage and recent *B. taurus* introgression in NA yak with increasing numbers of tySNPs. Our ultimate goal was to use a minimal set of tySNPs to accomplish multiple diverse genetic tests.

## Methods

### Ethics statement

This article contains no studies performed with animal subjects. The original sources of archived DNA samples used were either purchased from companies that collected them for artificial insemination and not for research (beef cattle), purchased or donated from individuals that collected them privately for their purposes such as food (bison and yak), or donated to the U.S. Meat Animal Research Center (USMARC) by private individuals that collected the samples privately for their own herd management purposes (beef cattle and yak, see *Acknowledgements*). The banteng DNA samples (1 ug each) were transferred for a fee under a Research Material Agreement that was executed on February 6, 2007 by the legal directors of the San Diego Zoo and the Technology Transfer Office of the USDA, ARS. The gaur blood samples were collected under Omaha Henry Doorly Zoo IACUC protocols on June 15,1999 by their Director of Animal Health, Dr. Douglas Armstrong, DVM in the presence of coauthor, MP Heaton. The animals were being evaluated for interstate transfer to another zoo which required a venous blood draw. A butterfly needle was used to fill blood tubes for required testing and DNA sample collection.

### Animals and WGS

**Figure 2.**
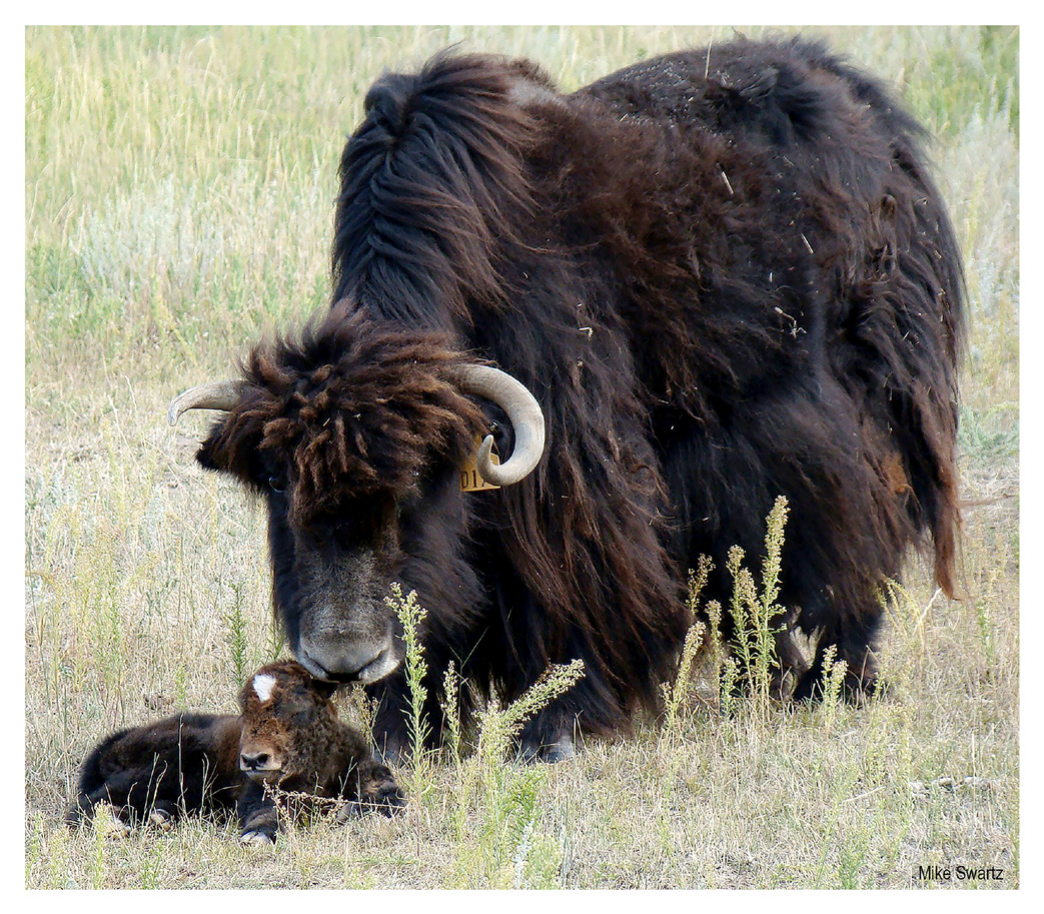
NA Reference yak Queen Allante and 2009 calf. (Figure2_QueenAllanteAndCalf600.TIF)

The NA reference domestic yak female used for WGS and alignment to the *B. taurus* genome was Queen Allante D171 (QA; Figure 2). QA died of natural causes in January of 2010 at approximately 30 years of age and, at the time of her death, was the oldest known living founder of the NA population. Fresh hide was collected post-mortem by the owner, frozen at -20°C, and shipped to USMARC for DNA extraction and production of genome sequences. Approximately 20-fold coverage of FASTQ files for QA were obtained from BioProject accession number PRJNA325061 (BioSample SAMN05558793, SRX2026482, SRX2026483 and SRX2026485 - SRX2026494). Details of library preparation and sequencing are as described by Heaton *et al*. ^21^. 

The Chinese reference domestic yak female used for alignment to the *B. taurus* genome was QH1 ^15^. Approximately 60-fold coverage of FASTQ files were obtained from BioProject accession number PRJNA74739 (BioSample SAMN00744358, SRX103173-SRX103192). This animal’s data set was originally used because it was the only other yak data set with sufficient coverage (>10X) for both variant discovery and accurate genotyping. Subsequently, additional yak data were available from BioProject accession number PRJNA285834, including *B. grunniens*: DYS74, SAMN03766772, SRX1056027; DYS77, SAMN03766773, SRX1056028; DYX12, SAMN03766774, SRX1056029; DYX11, SAMN03766775, SRX1056030; DYY31, SAMN03766776, SRX1056031, and *B. mutus*: WYX01, SAMN03766777, SRX1056032; WYX02, SAMN03766778, SRX1056033; WYX03, SAMN03766779, SRX1056034; WYX04, SAMN03766780, SRX1056035; and WYX05, SAMN03766782, SRX1056036 ^22^.

WGS and the haplotype-resolved F1 yak-cattle assembly of “Esperanza,” the calf of a female NA yak and a Highland beef bull ^17^, was also used for evaluating the accuracy of tySNPs ( ARS_UNL_BGru_maternal_1.0_p), BioProject accession numbers PRJNA551500 and PRJNA552915 (BioSample SAMN12153487,SRR12094761). In addition, research and commercial genotype data from 170 NA yak samples derived from 36 sources were used to estimate allele frequencies in a subset of 87 tySNPs.

The beef cattle panel consisted of 96 unrelated individuals from 19 popular U.S. beef breeds (USMARC Beef Diversity Panel version 2.9 [MBCDPv2.9]) ^21^. Pedigrees were obtained from leading suppliers of U.S. beef cattle semen and breed associations, and analyzed to identify unrelated individuals for inclusion. On the basis of the number of registered progeny, the breeds were estimated to represent greater than 99% of the germplasm used in the US beef cattle industry, contain more than 187 unshared haploid genomes, and allow a 95% probability of detecting any allele with a frequency greater than 0.016 ^23^. The breeds in MBCDPv2.9 were (in descending order of registered progeny circa 2000): Angus (n = 6), Hereford (n = 6), Charolais (n = 6), Simmental (n = 6), Red Angus (n = 6), Limousin (n = 6), Gelbvieh (n = 6), Brangus (n = 5), Beefmaster (n = 5), Salers (n = 5), Shorthorn (n = 5), Maine-Anjou (n = 5), Brahman (n = 6), Chianina (n = 4), Texas Longhorn (n = 4), Santa Gertrudis (n = 4), Braunvieh (n = 4), Tarentaise (n = 4), and Corriente (n = 4). The average genome coverage of FASTQ files for these 96 beef bulls was about 14-fold and is available in the NCBI SRA with links to BioProject accession number PRJNA324822 ^21^.

The other *Bos* species used consisted of approximately 10 to 14-fold coverage of WGS from gaur (n = 2), banteng (n = 2), and bison (n = 1) and were obtained from BioProject accession number PRJNA325061 including *B. gaurus*: 199911001, SAMN05558794, SRX2026439-SRX2026446, SRX2026451, SRX2026462, SRX2026473, SRX2026484, SRX2026495 - SRX2026498; 199911002, SAMN05558795, SRX2026447 - SRX2026450, SRX2026452 - SRX2026461, SRX2026463 - SRX2026464; *B. javanicus*: 200710001, SAMN05558796, SRX2026465 - SRX2026468; 200710002, SAMN05558797, SRX2026469 - SRX2026472; and *B. bison*: 199912001, SAMN05558798, SRX2026474-SRX2026481 ^21^.

### Read mapping and variant discovery

All sequence data used in this study were quality trimmed using TrimGalore (version 0.5.0) and mapped to the *Bos taurus* assembly, UMD3.1 ^24^, using the Burrows Wheeler Aligner (version 0.6.1) ^25^ module *aln*. The sam-formatted file was then converted to bam format and sorted with samtools (version 0.1.18) ^26^. Samtools was also used to mark the PCR duplicates with the “markdup” function. The Broad Institute’s Genome Analysis Toolkit ^27^ (GATK version 1.5-32-g2761da9) module “IndelRealigner' was used to ensure that any insertions or deletions were consistently aligned. Variant discovery and genotyping for mapped WGS datasets for yak, gaur, banteng, and bison animals (n = 7) were performed using the GATK module UnifiedGenotyper (version 3.4-46-gbc02625) run with genotyping_mode=DISCOVERY. The VCF file was filtered using a custom program ( ParseYakCoarse.java) ^28^ to identify tySNPs where the gaur, banteng and bison animals (n = 5) were homozygous for the same inferred *Bos* ancestral allele (e.g. Figure 1A, “C/C”), and both reference yaks were heterozygous for the *Bos* ancestral allele and the yak-associated allele (e.g. Figure 1A, “C/T”). The yak-associated allele was not present in the bovine UMD3.1 assembly or the other three *Bos* species, and the ancestral allele differed from the allele present in the UMD3.1 assembly

### Screening for a third common allele in beef cattle

The VCF file containing these above filtered SNPs was then used as the --alleles argument to the Unified Genotyper with genotyping_mode=GENOTYPE_GIVEN_ALLELES, and the 96 BAM files from the cattle diversity panel as the BAM input files. The “--intervals” option was also used with a .bed file that had a record specifying a locus of 2001 bases centered on each of the SNPs. This limited the scope of the genotyping to only the newly filtered SNPs and produced genotypes for all 96 beef cattle animals across those SNPs. A custom program ( ParseYakFine.java) ^28^ was written to filter the VCF record, passing only those records where 183 or more of the 192 possible beef cattle alleles (i.e. >95%) was a cattle-specific, bovine UMD3.1 reference allele (e.g., Figure 1A, “A/A”). More than half of the records in the resulting VCF file had a FILTER value of LowQual since no non-reference allele was found in any animal in those records. In order to use the resulting VCF as input for the next step this FILTER value was edited using the text editor emacs and “LowQual” was replaced with “.”. The edited file was used as the input for genotyping WGS.

### Genotyping tySNPs in wild and domestic Chinese yak

A VCF file containing candidate tySNPs was used as the --alleles argument in the Unified Genotyper software with genotyping_mode=GENOTYPE_GIVEN_ALLELES and the bam files created for five wild and four domestic Chinese yak were genotyped. The “--intervals” option was also used with a .bed file that had a record specifying a locus of 2001 bases centered on each of the polymorphisms in order to limit the scope of the genotyping to those loci relevant to this study. A custom program ( ParseVCF_OtherYak.java) ^28^ was written to count the number of ancestral, yak-associated and *B. taurus*-associated alleles present in each yak.

### Extracting adjacent conserved sequences, identifying neighboring SNPs, and masking

The *B. taurus* UMD3.1 chromosome position and alleles for the candidate markers were used with a custom program ( ParseYakFinal.java) ^28^ to extract 100 bases of flanking sequence both 3’ and 5’ of the marker position. The bam files for the five wild and four domestic Chinese yak, and the 96 beef cattle diversity panel ^21^, were used to identify neighboring SNPs that could disrupt heteroduplex formation with oligonucleotides used in genotyping assays on a variety of genotyping platforms. The GATK UnifiedGenotyper was run with genotyping_mode=DISCOVERY and the --intervals value was set as a .bed file containing records for all loci specified with 2001 bases centered on each marker. Cattle variants discovered in the 100 bp sequences adjacent to tySNPs on either side were replaced with “N” if their allele frequencies were greater than or equal to 5% (i.e., 9 or more of 192 possible alleles). These variant flags may be used by genotype assay design software to redirect oligonucleotide placement to more conserved flanking sequences. Similarly, yak variants flanking the tySNPs at any frequency (compared to the *B. taurus* UMD3.1 assembly) were also replaced with N for the same reason.

### Aligning tySNPs to yak genome for chromosome position and cluster assignment

The last step in filtering candidate tySNPs was aligning 200 bp of flanking sequence to the haplotype-resolved F1 yak assembly (ARS_UNL_BGru_maternal_1.0_p). This determined the yak chromosome and position for the tySNP. Markers were excluded from the group if they did not map exactly once to the assembly. The remaining tySNPs were manually assigned to marker “bins” based on their chromosome position. Clustered markers (e.g., less than 1 Mb) were typically grouped in the same bin to allow SNP assay design software choices for the most amenable target in the region. Where possible, the goal for spacing between bins was 5 Mb. With perfect 5 Mb spacing in the 2479 Mb yak autosomal genome, there would be about 496 bins plus 29 for the end bins.

### Matrix-assisted laser desorption/ionization time-of-flight mass spectrometry (MALDI-TOF MS) assay design for a subset of 87 tySNPs

Prior to assay development, the cutoffs for call rate (total genotypes obtained/total genotypes possible) and accuracy (correct genotypes/total genotypes obtained) were set at 97% and 99%, respectively. For the purposes of parentage exclusion the cutoff call rate of 97% means that a minimum of 94% of the tySNPs will have a genotype for each of the two animals in a pairwise comparison. Although these cutoffs are relatively high, we consider them to be the “gold standard” in SNP-based parentage testing and are within the capability of today’s DNA testing technology. Sets of parentage SNPs that meet these standards substantially increase the efficiency of testing ^9^. Assay development and genotyping was performed at Neogen Genomics (Lincoln, Nebraska, USA) with the MassARRAY platform and iPLEX GOLD chemistry according to the manufacturer’s instructions (Agena Bioscience, San Diego, California, USA). The multiplex assays were designed with the manufacturer’s assay design software and a preliminary set of 139 bins with 518 tySNPs aligned to the B. taurus UMD3.1 assembly. The software options were set for a maximum of 48 assays per plex and to select one tySNP marker available in any bin. Extension probe concentrations were adjusted empirically to optimize signal across the entire mass spectrum. The three multiplex assays were run on DNA from the NA reference yak (QA) and other NA yak. Specific SNP assays that produced low call rates or high error rates were censored from data sets. For the present report, genotype data was used for any of these 139 binned SNPs that passed the assay criteria above and had a unique mapping position in the haplotype-resolved F1 yak assembly.

### Estimating the probability of identity (P _I_) and probability of exclusion (P _E_) with tySNPs

P _I _is an estimate for the probability of a coincidental genotype match between two animals. Assumptions used for analyzing tySNPs included Hardy-Weinberg (HW) distributions of genotypes, a negligible frequency of the *B. taurus*-associated allele, and that the average MAF for yak parentage alleles in NA yak is representative. Briefly, the P _I_ for locus A with SNP alleles A _1_ and A _2_ was the sum of the squares of the three genotype frequencies: P _I_ =  (χ _11_) ^2 ^+ (χ _12_) ^2 ^+ (χ _22_) ^2^, where χ _11_, χ _12_, and χ _22_ were the relative genotype frequencies of A _1_A _1_, A _1_A _2_, and A _2_A _2_, respectively ^29^. The combined P _I_ for multiple SNP markers was the product of the P _I_ for each individual marker. The underlying assumption was that the marker spacing was sufficient for meiotic recombination to cause alleles to be randomly associated with one another. However, as parentage SNP density increases, the validity of this assumption is decreased. Thus, the combined P _I_ for more than one parentage SNP per chromosome is an underestimate of the probability of a coincidental match between random animals from the population, due to linkage disequilibrium between SNPs on the same chromosome. Also, for the purposes of estimating P _I_ and P _E_, we assumed that the frequency of the *B. taurus*-associated allele (A _3_) was negligible.

P _E_ is the probability that a random animal would be excluded from parentage. P _E_ is also the least complicated method of parentage analysis and estimates the fraction of potential adults excluded from parentage. In this report, all P _E_ estimates stringently used only one parent’s genotype information i.e., the most challenging scenario. Thus, exclusion was based only on the frequency of the opposing homozygous SNP genotypes in the offspring and the purported parent as previously described ^9^. Briefly, the probability of opposing homozygotes (P _OH_) between a random offspring and a random eligible adult at SNP locus A with alleles A _1_ and A _2_, was calculated as follows: P _OH_ = (χ _11_offspring)(χ _22_adult)+(χ _22_offspring)(χ _11_adult), where χ _11_ and χ _22_ were the relative genotype frequencies of A _1_A _1_ and A _2_A _2_, respectively for the adults or offspring groups. The frequencies of homozygous SNP genotypes were assumed to be the same within a breed group regardless of age. Thus, for a single biallelic SNP, P _E_ = P _OH_ = 2(χ _11_)(χ _22_) when one of the parent’s genotypes is unavailable. This represents the fraction of eligible adults that would be excluded from parentage at one locus, averaged over all comparisons between offspring and adults. Without using the other parent’s genotype information, the combined P _E_ for multiple SNPs was as follows: P _E(SNPn) _= P _E(SNP1) _+ R _1_P _E(SNP2) _+ R _2_P _E(SNP3)_ … + R _n-1_P _E(SNPn)_, where P represents the fraction of eligible adults excluded by the first SNP and R _1_ is the remaining fraction of unexcluded adults. R _2_ to R _n-1_ are remaining fractions of unexcluded adults after each round of subsequent testing with n parentage SNPs. Thus, for 29 parentage SNPs (one on each autosome), the combined P _E_ for unrelated parents is given by: P _E(29)_ = P _E(1)_ + R _1_P _E(2)_ + R _2_P _E(3)_ …+ R _28_P _E(29)_. As was the case with combined P _I_, the combined P _E_ for more than 1 parentage SNP per autosome is an underestimate of the probability that a random alleged parent would be excluded from parentage due to linkage disequilibrium between SNPs on the same chromosome. For related parents, the P _E_ for each SNP was multiplied by a coefficient of relatedness ( *r*), where *r* = 0.125, 0.250, or 0.500 [ 34]. Thus, P _E(29)_ for related parents  =  ( *r*P _E(1) _+ *r*R _1_P _E(2) _+ *r*R _2_P _E(3)_ … + *r*R _29_P _E(29)_).

### Identifying exclusions in a tri-allelic SNP system

Triallelic SNPs can lead to parentage exclusion in ways that biallelic do not, due to the presence of *B. taurus*-associated alleles in some individuals. Consider the three alleles of a tySNP: the *Bos* ancestral allele (A _1_), the yak parentage allele (A _2_), and the *B. taurus*-associated allele (A _3_). An exclusion occurs whenever the calf and the adult do not share an allele. Thus, when evaluating “one-parent” scenarios where the dam’s genotypes are unavailable, the analysis is not limited to only the opposing homozygous genotypes. Heterozygous sites may become informative when the calf or the adult possess a copy of the *B. taurus*-associated allele. Two common examples of these genotype configurations are when the calf is A _1_/A _1_ and the adult is A _2_/A _3_ genotype, or when the calf is A _1_/A _3_ and the adult is A _2_/A _2_. In both examples the adult is excluded from parentage. Similarly, when evaluating “two-parent” scenarios where the dam’s genotype at some sites can be used to determine the sire’s allele in the calf, a heterozygous site in the dam and the calf may become informative if one or the other possesses a copy of the *B. taurus*-associated allele. For example, when the calf is A _1_/A _2_ and the dam is A _2_/A _3_, the calf’s sire allele is A _1_ and will exclude all adults that do not carry the A _1_ allele. Although parentage exclusions caused by the *B. taurus*-associated alleles only occur in a few percent of the NA yak genotypes comparisons, it is important to account for them when processing parentage tests in commercial settings.

### Simulations for evaluating the power of exclusion with tySNPs in the presence of genotyping error

A triallelic SNP has an increased potential to exclude random adults from parentage compared to a biallelic SNP. However, the exceedingly low frequency of the *B. taurus*-associated allele in yak results in a negligible contribution to yak parentage exclusion. Consequently, the third allele was ignored for the purposes of these simulations. One million random offspring/adult pairings were simulated with tySNP allele frequencies inferred from genotypes of 170 NA yak with HW assumptions. The offspring/adult pair approximates the one-parent parentage testing scenario since the other parent’s genotypes were not used to phase the offspring’s heterozygous sites. An exclusion from parentage was counted for each tySNP site where the offspring and the adult were homozygous for different alleles. The frequency distribution for exclusions for a given set of tySNPs was determined by summing the exclusive sites for each offspring/adult pairing over all pairings. A second simulation was performed to test the effect of “allelic dropout,” a well-known source of genotyping error, and the systematic error we expect most often with a high-quality sample in this SNP panel. This occurs when other genomic SNPs are present in the binding sites for the three oligonucleotides used in a MALDI-TOF assay. These SNPs may disrupt heteroduplex formation in any of the three required assay primers and cause the linked target allele to be absent from the genotyped alleles. In this simulation, a yak sire was simulated by choosing alleles for each tySNP based on allele frequencies inferred from genotypes of 170 NA Yak with HW assumptions. For each tySNP, one of the sire yak alleles was assigned to the offspring with the second allele chosen at random based on the allele frequencies for the corresponding marker. A fixed genotype error rate was then applied to each genotype. For those genotypes chosen to be in error, one of the two alleles was omitted from the call, and the event was recorded if an artifactual exclusion was introduced.

### Simulations for evaluating the power of tySNPs to detect recent F1 hybridization events

**Figure 3.**
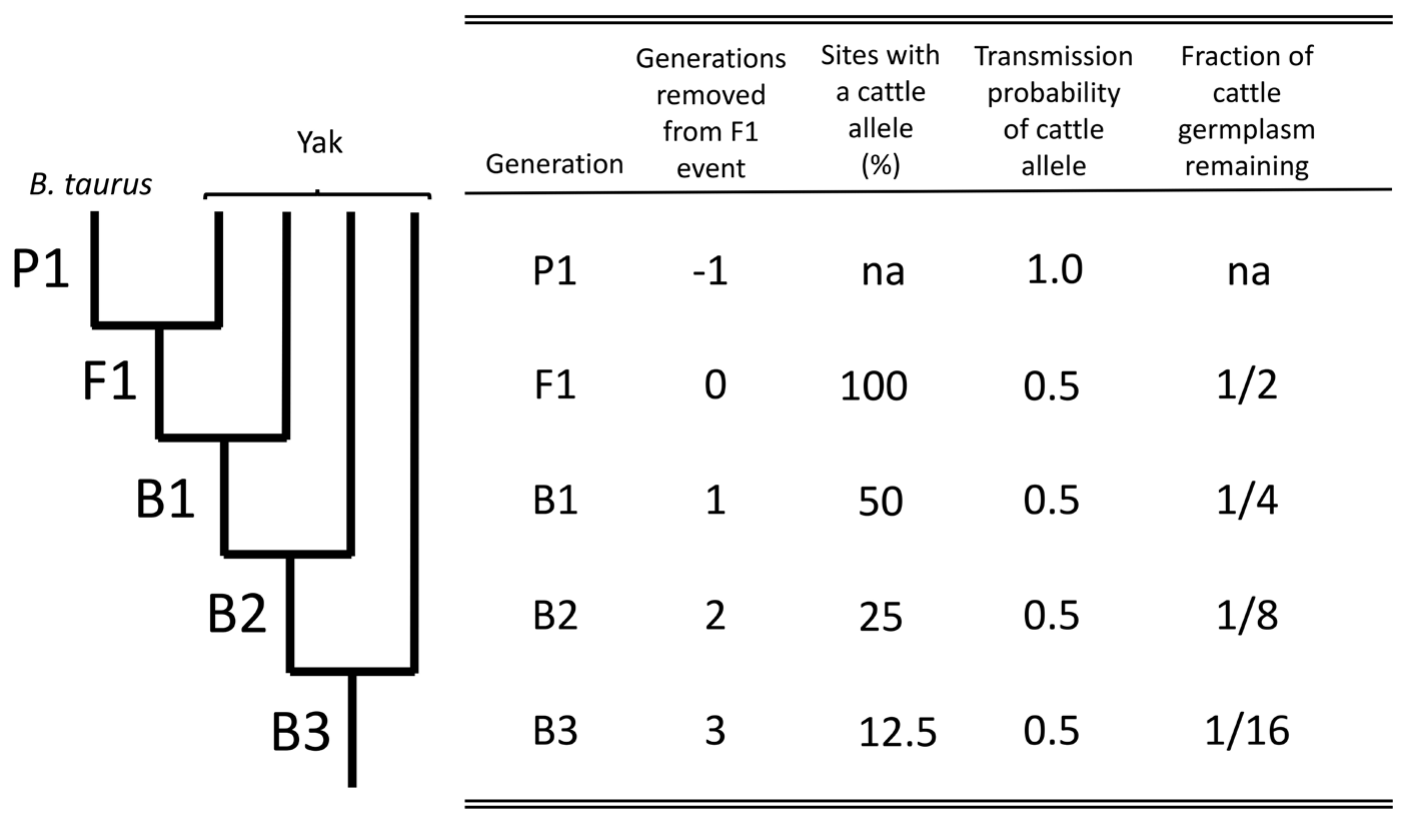
Detecting cattle alleles from an F1 hybridization event followed by yak backcrossing. Abbreviations: P1, parental generation; F1, hybrid generation; B, backcross generations; na, not applicable. (Figure3_CattleIntrogressionChart3.TIF)

An F1 yak/cattle hybridization event is readily detected with tySNPs since the offspring would have a *B. taurus*-associated allele at every site ( Figure 3). However, how many back crossings with yak can occur before the cattle alleles can no longer be reliably detected? The cattle allele transmission probability is 0.5 for every generation past the F1 event. Thus, the offspring would be expected to lose, on average, half of the *B. taurus*-associated alleles each subsequent generation ( Figure 3). This would result in approximately 50%, 25%, and 12.5%, in the first, second, and third generations, respectively, after the F1 event. A simulation was performed where an F1 yak/cattle cross had one cattle allele and either a *Bos* ancestral or yak-associated allele at each of tySNP positions. From that simulated F1 offspring, a cross was also simulated with a yak that had no cattle alleles at any tySNP. This was accomplished by randomly removing one of the two alleles for the F1 at each site and adding back a second non-cattle allele that would have been contributed by the backcross. Starting with the resulting genotypes from this simulated mating, a new set of genotypes was generated using the same process for an additional five generations. The distribution of the remaining cattle alleles per generation was subsequently plotted.

## Results

### Identification of candidate tySNPs

**Figure 4.**
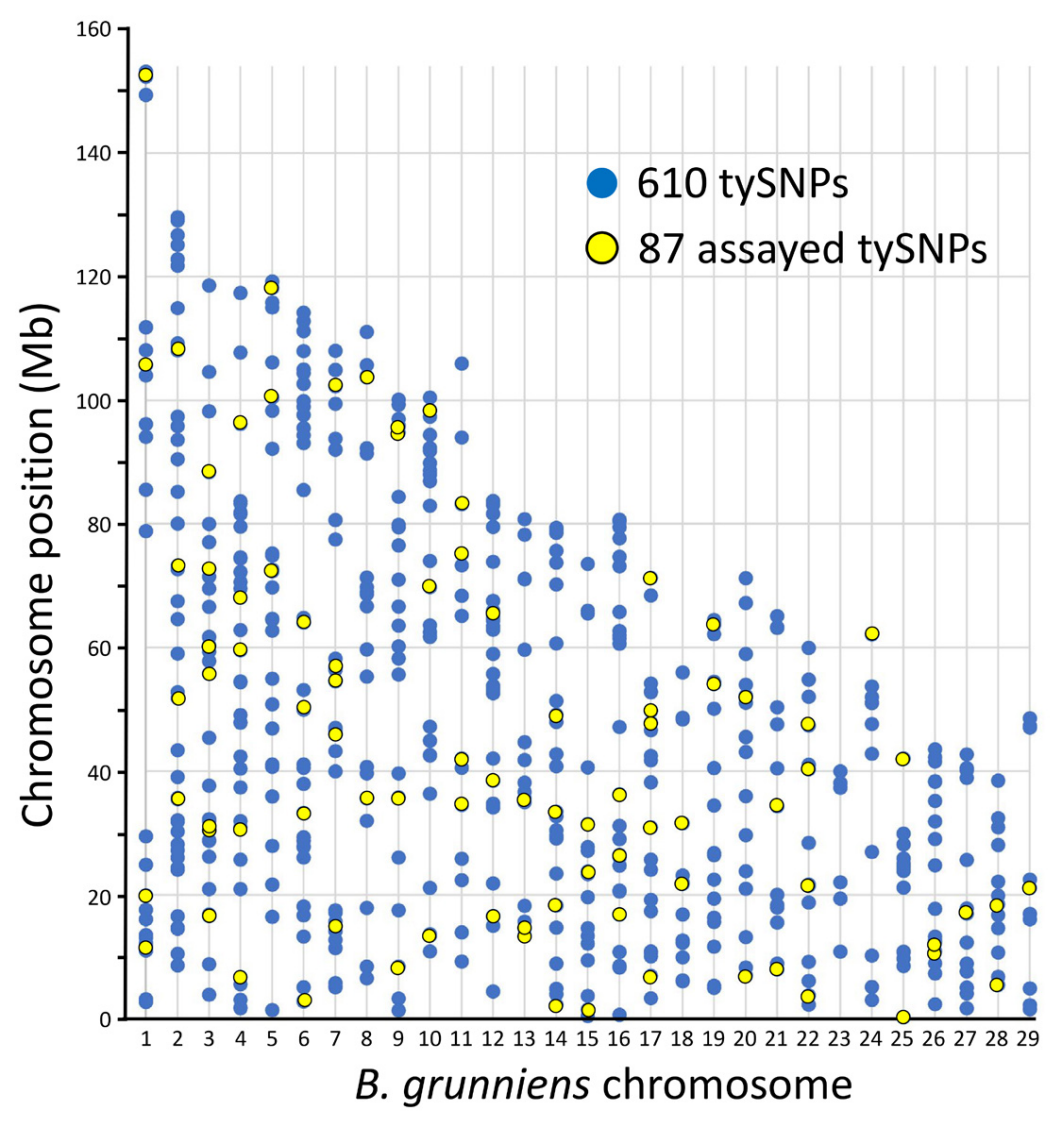
Chromosomal locations of tySNPs. The positions of 610 tySNPs are shown aligned to the ARS_UNL_BGru_maternal_1.0_p yak assembly. Yellow dots indicate those 87 tySNPs converted to assays and used in NA yak to estimate allele frequencies and utility. (Figure4_610tySNPs_442bins4.TIF)

Aligning the QA and QH1 reference yak genome sequences to *B. taurus* reference genome assembly identified 3133 candidate tySNPs that were: 1) heterozygous in both yak, 2) homozygous in gaur, banteng, and bison, and 3) had a third *B. taurus*-associated allele. Subsequent filtering of the less frequent cattle alleles (i.e., less than 0.95) reduced the set of tySNPs to 1023. Aligning the flanking sequences of the 1023 candidate tySNPs to the *B. grunniens* haplotype-resolved yak genome assembly identified 612 tySNPs with unique chromosome coordinate positions. Two additional candidate SNPs were removed for being monomorphic after all tySNPs were genotyped in 170 NA yak in a final round with updated statistics. The remaining 610 tySNPs were grouped into 441 regional clusters (i.e., bins) with an average distance of 5.26 Mb between bins (Table S1) ^30^. Of the 610 tySNPs, 87 coincided with an unpublished set of tySNPs for which we previously developed MALDI-TOF MS genotyping assays (Table S2, see *Methods*) ^31^. These 87 tySNPs were a subset of markers identified by the same process except for alignment to the haplotype resolved yak genome. Their prior selection predated the availability of this novel yak reference assembly. Thus, their unique alignment and yak genome positions relative to the yak reference assembly were previously unconfirmed. The positions of the 87 tySNPs, together with the other 523 tySNPs, are shown on the haplotype-resolved yak genome assembly ( Figure 4). Together, all 610 tySNPs, their bins, and sequence information are suitable for input into SNP assay design software for a variety of genotyping platforms.

### Allele frequencies of 87 selected tySNPs

**Figure 5.**
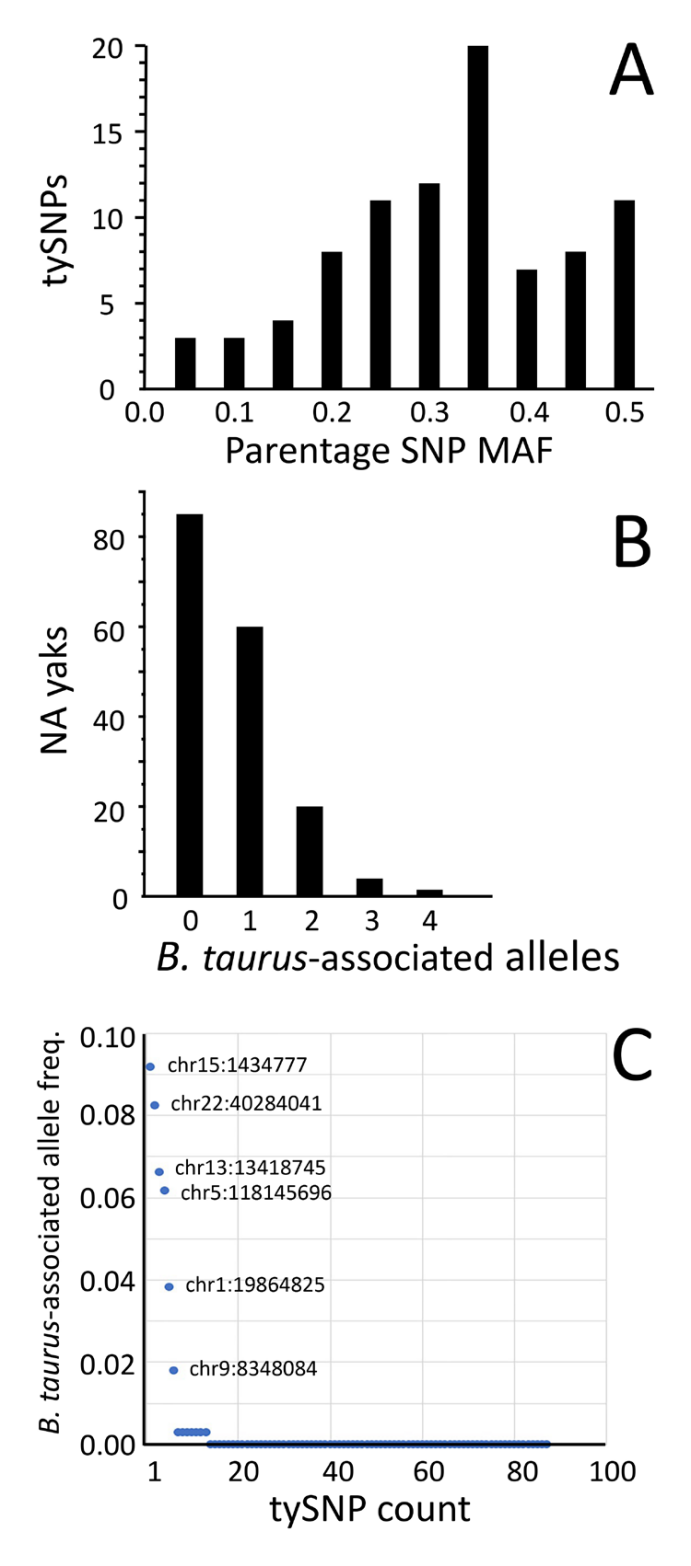
Allele frequency distributions of 87 tySNPs based on genotypes from 170 NA yaks. ( **A**) Yak parentage SNPs MAFs. ( **B**) *B. taurus*-associated alleles within animals. Panel **C**, variation in *B. taurus*-associated allele frequencies among different tySNPs. (Figure5_87_SNPs_MAFCatt4.TIF)

Genotypes from MALDI-TOF MS assays for the 87 tySNPs were scored in 170 NA yak from 36 sources to provide an estimate of the allele frequencies of parentage alleles and cattle alleles (Table S5) ^32^. The overall SNP genotyping rate (i.e. “call rate”) was 0.9952 for 87 tySNPs in 170 animals. The *Bos* ancestral allele was the major allele for most of the tySNPs in NA yak (67%). The average MAF for the yak parentage allele was 0.296 with 53% of them making the 0.30 cutoff ( Figure 5A). The overall *B. taurus*-associated allele frequency was very low (0.0043) with more than half of the NA yaks having zero of 174 possible cattle alleles among the 87 sites tested ( Figure 5B). The *B. taurus*-associated allele frequencies for six of the 87 tySNPs were higher than the rest of the group, although less than 0.1 overall. ( Figure 5C). 

### Animal identification and parentage exclusion with 87 SNPs

**Figure 6.**
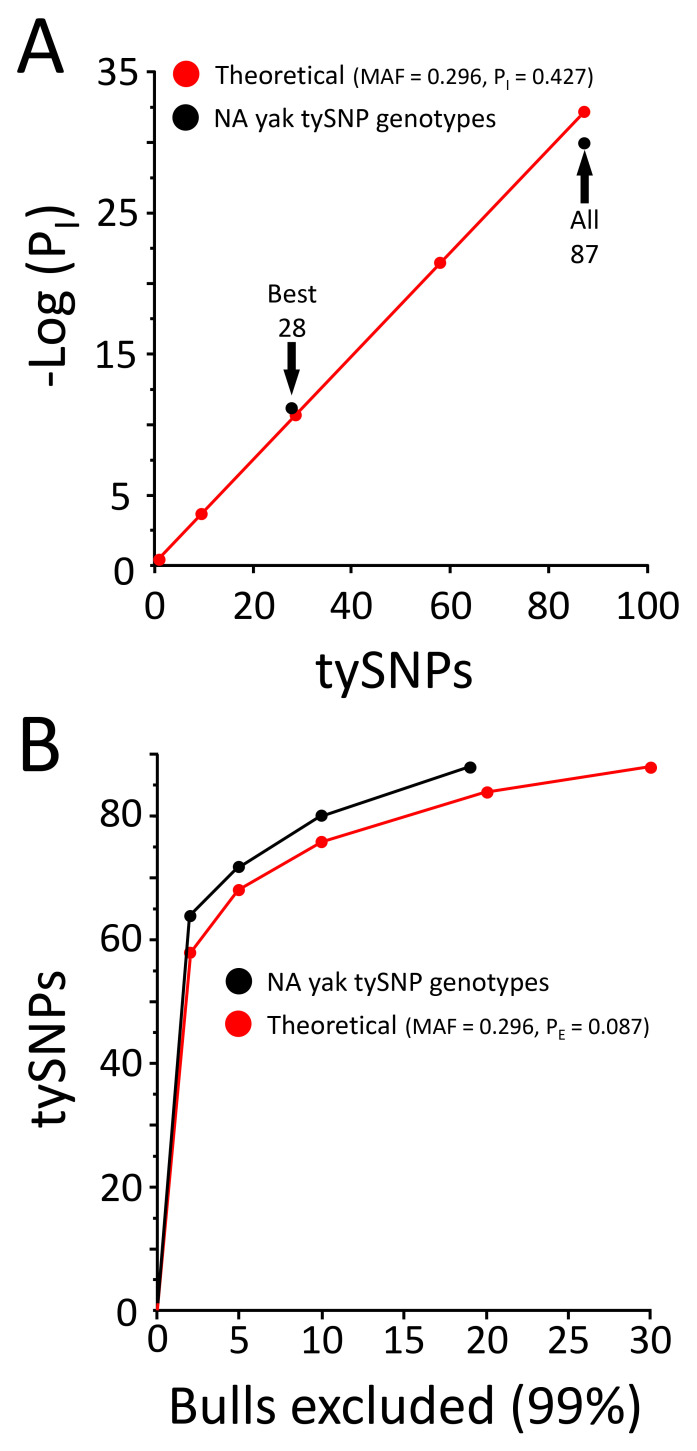
P _I_ and one-parent P _E_ estimates for tySNPs in NA yaks. ( **A**) The probability of a coincidental genotype match with tySNPs. ( **B**) The number of tySNPs needed to exclude 99% of the candidate bulls from parentage in the absence of the dam’s genotype information. (Figure6_PI_PE_7.TIF)

Using the average MAF for 87 tySNPs (0.296) and HW assumptions, the P _I_ for one SNP was 0.427, meaning that approximately 43% of NA yak would be expected to share identical genotypes at an average tySNP. Extending this to 29 tySNPs distributed equally on 29 autosomes yielded a combined theoretical P _I_ of 1.92 × 10 ^-11^. However, selecting the best high-MAF tySNPs gave a slightly better result with only 28 autosomes: P _I_ = 5.81 × 10 ^-12^ ( Figure 6A, Table S2) ^31^. For determining a calf’s sire without the dam’s genetic information (i.e., one-parent parentage testing), the theoretical P _E _for one SNP was 0.087 with the same MAF and HW assumptions. Thus, approximately 9% of candidate bulls can be excluded with one tySNP. To exclude a group of 30 candidate bulls at the 99% confidence level would require 87 tySNPs all with the same P _E_ ( Figure 6B). There were 19 fewer bulls excluded at the 99% confidence level when using the actual P _E_ calculated from NA genotypes for all 87 tySNPs, likely due to linkage between tySNPs and low MAFs in some markers. By adding the dam’s genotypes, the power of exclusion approximately doubles due to the ability to phase the calf’s heterozygous alleles. 

### Allele distributions of 610 tySNPs genotyped *in silico* with WGS and comparison to the selected 87 SNPs

The intrinsic properties and allele distributions of the 610 tySNPs were further evaluated *in silico* with WGS from yaks, *Bos* species, beef cattle and an F1 yak-cattle hybrid trio. By design, the two reference yak were heterozygous at all 610 sites, having exactly one *Bos* ancestral allele and one yak-associated allele ( Table 1). Excluding monozygotic twins, these are the only two yaks expected to have all 610 identical heterozygous genotypes, since the tySNP marker screening was targeted to them. Also by design was the *Bos* ancestral allele frequency being fixed in gaur, banteng, and bison due to selection for this property in the filtering. A notable exception was a single tySNP in bison that was heterozygous for a 4th allele at one site (A/T, ARS1.2-UCD chr12:83562937, Table S2) ^31^. An unexpected genotype result was found in one of the five Chinese domestic yak data sets (DYY31, SAMN03766776), which contained greater than 98% *B. taurus*-associated alleles. Additional analyses performed on its mapped WGS dataset (Table S6) ^33^ confirmed this to be a *B. taurus* data set and it was eliminated from subsequent analyses. 

**Table 1.** Allele frequencies of 610 tySNPs in WGS data sets.

			Alleles		
Animal or group	No.	WGS call rate (ave.)	*Bos* ancestral	Yak- associated	Cattle- associated
NA ref. yak (QA)	1	1.0000	0.5000	0.5000	-
Chinese ref. Yak (QH1)	1	1.0000	0.5000	0.5000	-
Gaur	2	1.0000	1.0000	-	-
Banteng	2	1.0000	1.0000	-	-
Bison	1	1.0000	1.0000	-	-
Chinese wild yaks	5	0.9790	0.6237	0.3721	0.0042
Chinese domestic yaks	4	0.9762	0.6076	0.3836	0.0076
Beef cattle	96	0.9997	0.0104	0.0028	0.9865
Highland bull (sire)	1	0.9984	0.0016	-	0.9984
NA yak (dam)	1	1.0000	0.5459	0.4443	0.0082
F1 yak-cattle hybrid	1	1.0000	0.2738	0.2213	0.5049

The remaining Chinese yak data sets were analyzed for ancestral allele and cattle allele content. The average ancestral allele frequency was slightly higher in wild yaks (0.624) compared to domestic yaks (0.608), while the *B. taurus*-associated allele frequencies in these yaks were lower (0.0042 and 0.0076, respectively, Table 1). These *B. taurus*-associated allele frequencies in Chinese yaks are compared to 0.0043 estimated for 87 tySNPs genotyped in NA yaks. In beef cattle, the *Bos* ancestral allele frequency was only 0.010 due to the selection of *B. taurus*-associated alleles in the filtering process. The frequency of *B. taurus*-associated alleles in the 610 tySNPs was 0.9865 in beef cattle. These *B. taurus*-associated allele frequencies were consistent with WGS genotypes from the F1 yak-cattle hybrid family trio: 0.9984 for the Highland sire, 0.0082 for the NA yak dam, and 0.5049 for the F1 calf. Thus, the intrinsic properties and allele distributions of the 610 tySNPs genotyped *in silico* with WGS were consistent with those obtained from multiplexed MALDI-TOF MS assays for 87 tySNPs in the group of 170 NA yaks from 36 sources.

### Simulating parentage exclusion with larger sets of tySNPs

**Figure 7.**
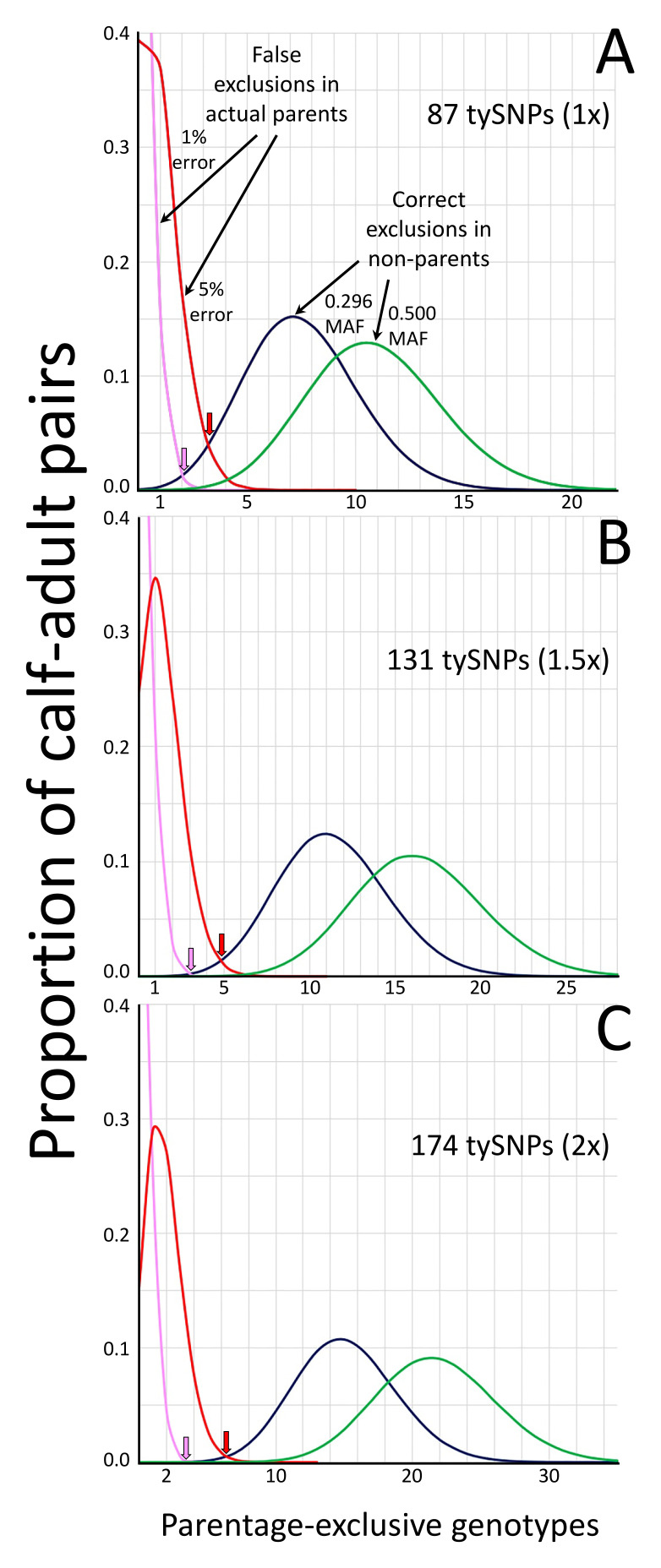
Simulated power for parentage exclusion with expanded tySNP sets, genotyping errors, and without the dam’s genotypes. Simulations were run as described in the Methods. Red and pink arrows point to the ambiguous overlap between correct exclusions in non-parents and the false exclusions in the actual parents at 5% and 1% genotyping error rates, respectively. (Figure7_ParentageSim4.TIF)

Based on the intrinsic properties of the 87 tySNPs above, it is reasonable to extrapolate their performance in scenarios with larger sets of tySNPs. Efficient parentage exclusion depends on a number of factors including: the MAF, the number of markers, and the genotyping error rate. It also requires the majority of random non-parents to have significantly more exclusions (i.e, opposite homozygous genotypes) than the erroneous exclusions in the true parent due to genotyping errors. With the current set of 87 tySNPs and a 0.296 average MAF, most random calf-adult pairings had 5 to 10 exclusive genotypes (i.e., opposing homozygous genotypes) while most calf-parent pairs have only 0 to 2 false exclusive genotypes at a 1% genotyping error rate ( Figure 7A). At a 5% genotyping error rate, approximately 0.05 of the true parents are expected to have three false genotype exclusions and fall into the overlap with 0.03 of the correct exclusions in non-parents. This means that 0.0015 of the calf-adult pairs would be difficult to exclude from parentage due to the overlap between false exclusions in true parents and the correct but few exclusions in non-parents. This overlap can be improved by either increasing the number of similar tySNPs, using markers with higher MAFs, or reducing the genotyping error rate ( Figure 7B and 7C). In addition, if the dam’s genotypes are available for the same tySNPs, the power is essentially doubled.

### Power for detecting recent cattle introgression

**Figure 8.**
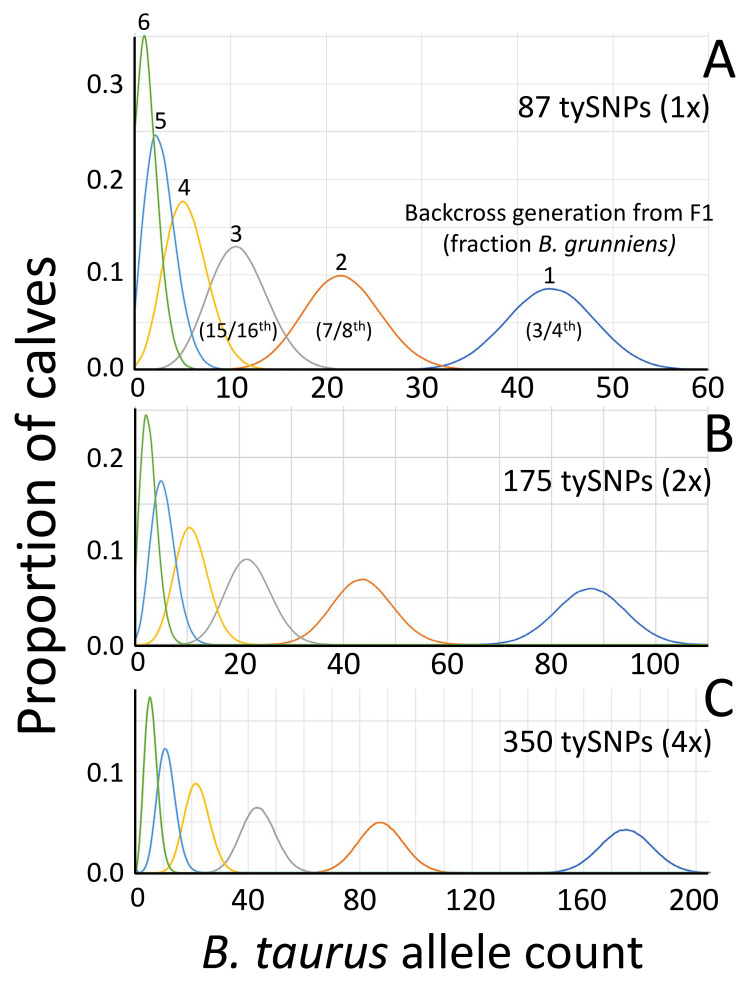
Simulated detection of cattle introgression with tySNPs. ( **A**), ( **B**) and ( **C**) Simulated results with 87, 174, and 350 tySNPs, respectively. Each peak represents one backcross generation removed from the F1 hybridization event. (Figure8_tySNP_BackCrossSim7.TIF)

The power to detect recent cattle introgression after F1 hybridization and subsequent yak backcrossing was simulated with sets of tySNP. Approximately half of the cattle alleles of an F1 hybrid are lost in subsequent backcross generations and thus fit a binomial distribution model. With 87 tySNPs, a third-generation backcross animal (i.e., 15/16th yak) would be expected to have 10 *B. taurus*-associated alleles detected ( Figure 8A). This is compared to a fullblood NA yak which had, on average, less than 1 *B. taurus*-associated allele per animal ( Figure 5B). Doubling the tySNPs increases the detection level to another backcross generation ( Figure 8B and 8C). The modes and shapes of these curves would be complicated and more numerous if there was more than one cattle/yak cross in a yak’s recent pedigree. Regardless, a NA yak with less than 3 of 87 *B. taurus*-associated alleles would be unlikely to have an F1 hybrid as a parent, grandparent or great grandparent.

## Discussion

Our aim was to identify a set of novel triallelic SNPs. We identified a novel set of 610 tySNPs where each marker has two alleles for NA yak parentage testing, and a third allele for identifying recent cattle introgression. Assay design data for these markers are provided in Table S1 ^30^. In addition to allele frequencies, the markers were stringently selected for reduced negative attributes such as indels, repetitive sequences, and flanking SNPs, to enhance their performance on present and future genotyping technologies. The tySNPs were distributed across the genome in 441 clusters (bins) with an average spacing of 5 Mb, based on the recently completed NA yak genome assembly ^17^. A subset of 87 tySNPs was developed for a MALDI-TOF MS platform and their abilities to: 1) provide each animal with a unique genetic identifier, 2) exclude random adults for parentage determination, and 3) identify animals with F1 hybridization events in their recent ancestry. By selecting the best tySNPs on each available autosome, only 28 tySNPs of the 87 were needed to uniquely identify every NA yak (estimated combined P _I_ = 5.81 × 10 ^-12^). Excluding monozygotic twins, this result means the odds of any two random NA yaks having the same genotypes at all 28 sites by chance would be 1 in 172 billion. This is enough power for tracing yaks and their products in the global food chain if needed. 

The power to exclude random adults from parentage with 87 tySNPs was high. Most random adults had between 5 to 10 tySNP sites that excluded them from parentage with a given offspring, i.e., did not share an allele with the offspring at those sites. However, the false exclusions in a true parent due to genotype error was only about 1 in 87 tySNPs with a genotype platform error rate of 1%. More than 99% of random adults can be excluded from parentage even with a genotyping error rate as high as 5% (i.e., four exclusions allowed in a true parent). Thus, most yak calves can be assigned to a single parent without having the other parent’s genotypes available. For multi-sire pasture mating situations this can reduce the cost of parentage testing by nearly 50%. Note that these estimates are for parentage exclusion with unrelated adults. The power to exclude full sibs from parentage is essentially reduced by half and thus may require use of the dam’s genotypes and/or more accuracy in the genotyping system ^9^. When 1000 offspring are involved, one effective strategy is to first assign the high-confidence calf-sire relationships with one-parent testing, and then use the dam’s genotypes to confirm sire exclusions on any remaining ambiguous calf-sire relationships.

The *B. taurus* species introgression in 170 NA yaks from diverse sources was low and not distinguishable from that of other yaks. With 87 tySNPs, no significant differences were detected between the average *B. taurus*-associated allele frequency in 170 NA yak (0.0042) and that of nine domestic and wild chinese yak (0.0057). This suggests the genetic foundation of the NA yak population overall is not significantly influenced with introgressed *B. taurus* germplasm. A low background of cattle introgression in yaks facilitates the ability of tySNPs to detect F1 hybridization events in the recent ancestry of a yak. With 87 tySNPs, simulations predicted confident detection of F1 hybridization events in yak containing as little as 1/16th *B. taurus* germplasm (i.e., three backcross generations after the F1 event). This would be sufficient to verify the accuracy of three- or four-generation pedigrees. It should be noted, however, that tySNPs are not informative with regards to which *B. taurus* animal was involved in the F1 hybridization event, because nearly all *B. taurus* animals are homozygous for the *B. taurus*-associated allele. We also noted that six tySNPs had *B. taurus*-associated allele frequencies between 0.01 and 0.10 in NA yak, while the *B. taurus*-associated allele frequencies in the other 81 tySNPs were essentially zero. Reasons for this could include: ancient *B. taurus* introgression and selection and/or the existence of both alleles prior to speciation. A future tySNP filter to consider would be less than 1% prevalence of *B. taurus*-associated alleles in NA yaks. In spite of these exceptions, overall *B. taurus*-associated alleles in tySNPs were rare in NA yaks, and thus able to identify animals descended from backcrosses of recent F1 hybrids.

The primary areas for improving the multiplexes set of 87 tySNPs include: marker abundance, parentage MAF, and genomic distribution. The 87 tySNPs presented here were developed out of necessity, prior to the availability of the NA yak genome assembly, and without prior knowledge of their allele frequencies in NA populations. While only 28 tySNPs provide more than enough power for genetic fingerprinting, additional tySNPs would add significant power for parentage exclusion. Using future WGS from an additional 10–15 NA yak would allow MAF estimates to be confidently assigned for the 610 tySNPs presented here. With this NA yak allele frequency information in hand, choosing tySNPs with the highest MAFs and increasing the number of markers to at least 131 (i.e., 1.5x) would increase the proportion of random adults excluded from parentage, as simulated in Figure 7B. This would also help with excluding highly related animals from parentage, for example, when full-sib bulls are simultaneously exposed to cows. Having a set of 131 tySNPs with high MAF would also somewhat improve the ability to detect F1 hybridizations events. Simulations showed that to gain one more generation in sensitivity for F1 hybridization detection, 175 (i.e., 2x) tySNPs would be needed. As genotyping technologies improve and become more cost-efficient, it may be possible to incorporate additional markers from the 421 bins of 610 tySNPs.

Triallelic SNPs have been systematically identified in other mammals. In *Homo sapiens*, a set of 1,270 polymorphic tri-allelic SNPs mined from the 1000 Genomes Project was recently used in forensic identification of missing persons ^34^. Interestingly, there are approximately twice as many triallelic sites in humans as expected by chance ^35^. It is conceivable that at least some of these may be due to introgression of archaic species into modern humans ^36^. Regardless of their use in humans, the multipurpose triallelic SNP approach presented here may be useful in other species that can hybridize, including bovids, cervids, odontids, camelids, equids, canids, felids, and ursids. An obvious immediate application is in NA plains bison ( *B. bison*). Plains bison are wild animals native to NA, have experienced a population bottleneck, hybridize with *B. taurus*, and farmed for their meat and byproducts. Ideally, WGS from a diverse group of 15 unrelated plains bison sampled from the NA herd would be used, together with the banteng, gaur, yak, and cattle to identify suitable triallelic bison SNPs (tbSNPs). By using WGS from 15 bison, the MAF estimates for bison parentage would be known *a priori* and could be more effectively used in the tbSNPs filtering process. Depending on the aims, WGS from NA wood bison ( *B*. *bison athabascae*) or European wisent ( *B. bonasus*) could also be included in filtering for MAF estimates and extend the potential utility of a tbSNP panel.

## Conclusion

Results from novel tySNPs presented here demonstrate that one minimal set of markers can be efficiently and accurately used for animal identification, parentage determination, and detecting recent F1 hybridization events to support herd management and breeding decisions of yak producers. The 610 tySNPs, their assay design information, multiplex MALDI-TOF MS assays for the subset of 87 tySNPs, and all other associated information are available for world-wide use without restriction.

## Data availability

### Underlying data

NCBI BioProject: *Bos mutus* strain:yakQH1 Genome sequencing and assembly. Accession number PRJNA74739.

NCBI BioProject: *Bos grunniens* Genome sequencing. Accession number PRJNA285834.

NCBI BioProject: Phased trio assembly of yak and cattle genomes. Accession number PRJNA551500.

NCBI BioProject: Bos grunniens x Bos taurus Genome sequencing and assembly. Accession number PRJNA552915.

NCBI BioProject: WGS data from diverse types of U.S. cattle. Accession number PRJNA324822.

NCBI BioProject: WGS data from Cetartiodactyla. Accession number PRJNA325061.

### Extended data

Figshare: Table S1. Genomic locations and sequences features of 610 tySNPs in 441 bins. https://doi.org/10.6084/m9.figshare.12472925.v1 ^30^.

Figshare: Table S2. Genomic locations and sequence features of a subset of 87 tySNPs. https://doi.org/10.6084/m9.figshare.12473087.v3 ^31^.

Figshare: Table S3. In silico genotypes derived from WGS for 610 tySNPs in yak, cattle, and other Bos species. m. https://doi.org/10.6084/m9.figshare.12473360.v1 ^37^.

Figshare: Table S4. Oligonucleotide sequences for MALDI-TOF MS assays of 87 tySNPs. https://doi.org/10.6084/m9.figshare.12473492.v1 ^38^.

Figshare: Table S5. MALDI-TOF MS genotypes of 87 tySNPs for 170 NA yak. https://doi.org/10.6084/m9.figshare.12473537.v1 ^32^.

Figshare: Table S6. Analysis of Chinese domestic yak WGS data set DYY31 for B. taurus sequences. https://doi.org/10.6084/m9.figshare.12682331.v1 ^33^.

## Software availability

All custom software used to analyze data for this project are available from the GitHub page for this project: https://github.com/kalbflei/YakParentageAndIntrogression.

Archived software at time of publication: https://doi.org/10.5281/zenodo.3988457 ^28^.

License: MIT license.
