# Supplementary material for: Using triallelic SNPs for determining parentage in North American yak ( Bos grunniens) and estimating cattle ( B. taurus) introgression
Source: F1000Res. 2020 Oct 29;9:1096. Originally published 2020 Sep 4. [Version 2] doi: 10.12688/f1000research.25803.2 (PMC7605211; doi:10.12688/f1000research.25803.2)
Supplement: Peer review history

# Peer review: Reviewer response for version 2

**DOI:** 10.5256/f1000research.30280.r73913

**Published:** 2020-10-30

**Authors:** Leslie A. Lyons

**Affiliations:** 1 Department of Veterinary Medicine and Surgery, College of Veterinary Medicine, University of Missouri, Columbia, MO, USA

Reviewer's concerns have been addressed - no further comment.

Is the rationale for developing the new method (or application) clearly explained?

Yes

Is the description of the method technically sound?

Yes

Are the conclusions about the method and its performance adequately supported by the findings presented in the article?

Partly

If any results are presented, are all the source data underlying the results available to ensure full reproducibility?

Partly

Are sufficient details provided to allow replication of the method development and its use by others?

Partly

Reviewer Expertise:

Genetics, DNA profiling, development of parentage, forensic and population DNA panels

I confirm that I have read this submission and believe that I have an appropriate level of expertise to confirm that it is of an acceptable scientific standard.

# Peer review: Reviewer response for version 1

**DOI:** 10.5256/f1000research.28475.r70891

**Published:** 2020-09-28

**Authors:** Oliver A. Ryder

**Affiliations:** 1 San Diego Zoo Institute for Conservation Research, San Diego Zoo Global, Escondido, CA, USA; 2 Department of Evolution, Behavior, and Ecology, Division of BZiology, University of California, San Diego, San Diego, CA, USA

The authors have used available genomes from wild and domestic yaks and related bovids to identify SNPs that are informative for partentage analysis in yaks, for which cattle have a nearly fixed alternate allele. Using available information on allele frequencies they estimate the power for parentage, even without data from one parent and introgression detection and conduct simulations to evaluate the number of SNPs required to reach different confidence levels for the two analyses.

 The paper is well written, highly detailed, and methods are adequately described.

Is the rationale for developing the new method (or application) clearly explained?

Yes

Is the description of the method technically sound?

Yes

Are the conclusions about the method and its performance adequately supported by the findings presented in the article?

Yes

If any results are presented, are all the source data underlying the results available to ensure full reproducibility?

No source data required

Are sufficient details provided to allow replication of the method development and its use by others?

Yes

Reviewer Expertise:

population management, comparative genomics

I confirm that I have read this submission and believe that I have an appropriate level of expertise to confirm that it is of an acceptable scientific standard.

# Peer review: Reviewer response for version 1

**DOI:** 10.5256/f1000research.28475.r70890

**Published:** 2020-09-23

**Authors:** Leslie A. Lyons

**Affiliations:** 1 Department of Veterinary Medicine and Surgery, College of Veterinary Medicine, University of Missouri, Columbia, MO, USA

The manuscript by Kalbfleisch *et al.*, explores the identification and use of triallelic SNPs (tySNPs) that are diagnostic for North American yak and cattle. The markers could then be used for population and parentage studies to manage the NA yak populations, attempting to keep inbreeding at a minimum and reducing introgression from cattle breeds. Sequence and assembly data from cattle (UMS3.1) and the F1 yak assembly were used to identify unique candidate tySNPs that had conserved flanking sequence to ensure robust amplification in both species and were dispersed across the genome. Overall, 610 autosomal tySNPs were identified. Allelic frequencies were determined for 87 tySNPs by genotyping in 170 NA yak. By simulations, the power for unique identification, parental exclusions and introgression up to three backcross generations from the F1 were estimated. A unique DNA profile could be obtained with 27 SNPs and the parentage and hybridization determinations required up to 87 SNPs.

 Current SNP-based parentage panels for cattle and other species contain over 100 to several hundred SNPs for unique identification and parentage determination. The panels need to be robust and valid for use in a variety of inbred breeds for a given species. Although the global population of domesticated yak is large and diverse, NA yak have a small population derived from founders in zoo populations imported from Europe. The herd has not been diversified using artificial insemination techniques. Ancient hybridization suggests 2 – 3% *Bos taurus* introgression. Recent, planned and unplanned cattle introgression has occurred in the yak NA population.

 The manuscript is well written and easy to understand and read. The authors provide extensive background in each section for the reader, however, some of which can be deleted to shorten the manuscript, but likely not a concern for the online publication. The study design is well planned and the bioinformatic approaches are sound. However, in the end, 87 SNPs were selected – but apparently not by the methods presented here. The presentation gets to ~610 candidate SNPs, where these 87 are amongst those candidates, but, the 87 were selected before these methods were conducted. So, how were the 87 SNPs initially selected and where are the other SNPs that likely failed in the development process? The 87 SNP panel also needs to be adjusted. This is a commercial set of SNPs that I fear are being promoted – not considering all the criteria even presented in this publication.

Introduction:

- The background information is very informative, but perhaps the introduction is a bit too long.
- What is the calculated evolutionary distance (time) between cattle and the yak? 
- Both Figure 1A and 1B are not necessary.  Perhaps use Figure 2 as Figure 1B?

Methods:

- Sample collection – “purchased or donated from zoological parks in their normal course of caring for animals”. Most zoological parks have ACUC protocols and require “study protocol forms” for samples released – even after opportunistic collections during health examinations. Perhaps the protocols should be confirmed?
- Can more of the Methods be shortened by using references? Such as details on the identity of the animals used for sequencing?
- The use of the pedigrees to select a strong representation of the beef cattle breeds is a strong aspect of the project.
- Correct spacing: “TrimGalore (version 0.5.0)and”
- Perhaps a supplementary table could be used to represent the information in the text regarding the number of each breed and the genome accession numbers, thus, shortening the text.
- “ *the cutoffs for call rate (total geno* *-types obtained/total genotypes possible) and accuracy (correctgenotypes/total genotypes obtained) were set at 97% and 99%, respectively.”*
  and
  “For the purposes of parentage exclusion the cutoff call rate of 97% means that a minimum of 94% of the tySNPs will have a genotype for each of the two animals in a pairwise comparison”
  These are stringent cutoffs for the data and should produce a very robust panel of SNPs.
- Used MALDI-TOF mass spectroscopy for genotyping: a preliminary set of 139 bins with 518 tySNPs aligned to the B. taurus UMD3.1 assembly. 48 assays per plex using one SNP per bin – producing 3 multiplexes.
- Although very thorough – can the section including the “Estimating the probability of identity (PI) and probability of exclusion (PE) with tySNPs”, and “Identifying exclusions in a tri-allelic SNP system” be shortened by referencing?
- Overall, the bioinformatic approaches appear to be sound and robust for this study.
- Figure 3 is not really needed.

Results:

- Interesting – “ *Of the 610* *tySNPs, 87 overlapped an unpublished set of tySNPs for which we previously developed MALDI-TOF MS genotyping assays (Table S2, see Methods)* *31*.” Thus, this panel was already developed and in use, but then verified by phased yak genome. Therefore – how were these original 87 tySNPs identified and designed?
- If starting from scratch with 610 tySNP candidates and then designing mass array multiplexes – 441 bins with 48 SNPs per multiplex = 10 assays minimum.
- Thus – it does not seem like the authors used the presented information to produce the 87 tySNP panel. After the fact – they are validating the panel but not suggesting any improvements from the data produced by the methods performed in the manuscript. For example, at least 5 or so of the 87 tySNPs appear to be very physically close in Figure 4 – and hence should not be in the panel.
- The *in silico* analysis for the 610 SNP allele frequencies confirmed the allelic frequencies for the 87 SNPs that were genotyped in the 170 yaks. However, could a better set of tySNPs have been selected from this data?
- All the exclusion and introgression calculations are based on the 87 SNPs.
- No animals with documented, known hybridization were genotyped for validation.

Discussion:

- *“Our aim was to identify a minimal set of novel triallelic SNPs to accomplish multiple diverse genetic tests in NA yaks.”*
  The authors do define a robust set of triallelic SNPs – however – the most robust or minimal set has NOT been identified because this paper confirms an *a priori* set of 87 SNPs that were selected using perhaps a different process as described here. The authors need to confirm the same process was used to select the 87 SNPs – short of using the phased yak assembly for confirmation and positioning, which they indicate.
- This manuscript does indicate the 87 SNPs developed for yak testing are robust and have good exclusion rates, but it does not select the best SNPs from the 610 to produce the panel. No modifications are suggested for the panel – including for SNPs now shown to be in close proximity or the 6 tySNPs with *B. taurus* – associated allele frequencies. However, the authors do suggest that better SNPs could be selected in more yak WGS data was available. Another way to produce this data would be to make additional multiplexes from the remaining 610 SNPs and genotype the same 170 yaks. If 87 SNPs had already been produced – likely – additional data on tySNPs not selected for the panel is likely available. 
- In the end, the NA yak are not significantly influenced with *B. taurus* introgression, thus, a standard set of SNPs would be likely sufficient, although introgression could not be monitored.

Is the rationale for developing the new method (or application) clearly explained?

Yes

Is the description of the method technically sound?

Yes

Are the conclusions about the method and its performance adequately supported by the findings presented in the article?

Partly

If any results are presented, are all the source data underlying the results available to ensure full reproducibility?

Partly

Are sufficient details provided to allow replication of the method development and its use by others?

Partly

Reviewer Expertise:

Genetics, DNA profiling, development of parentage, forensic and population DNA panels

I confirm that I have read this submission and believe that I have an appropriate level of expertise to confirm that it is of an acceptable scientific standard, however I have significant reservations, as outlined above.

# Response

**Published:** 2020-10-15

**Authors:** Ted Kalbfleisch

**Affiliations:** University of Kentucky, USA

Dear Reviewer 1,

     Thank you for your detailed comments on our manuscript.  Regarding the concern surrounding the 87 markers used, we revised the manuscript to highlight our aim of showing the variety of purposes to which triallelic yak SNPs could be applied, as our intention was not to validate a livestock SNP panel.  We regard the identification of the 610 tySNPs and their genomic metadata in Table S1 as a significant outcome of the present report.  We can confirm the subset of 87 tySNPs were identified by the same process as the 610 tySNPs, except for the use of the haplotype-resolved Yak reference genome for determining their correct and unique genome positions.  Like all the candidate tySNPs, their MAFs were unknown at the time of their original selection.  These 87 tySNPs were also identified a second time in the group of 610 as they met all the same selection criteria.  None of the 610 tySNPs were excluded for being close to another tySNP (hence binning), since automated assay design software needs options.  We have updated the new version of the manuscript as described below to highlight these facts.

With respect to the comment “This is a commercial set of SNPs that I fear are being promoted – not considering all the criteria even presented in this publication.”  All of the genotype data presented here were produced for research purposes and predate any commercial offering of a yak tySNP test.  The data were meant to provide a statistically significant sampling of the intrinsic properties expected in 170 NA yak for the 610 markers identified here.  Although a commercial tySNP test is now available and contains the 87 tySNPs, we do not endorse it to the exclusion of other markers or test panels that may be suitable.

**Page 8, column 1, paragraph 1, line 13 of the first version:**

**“Of the 610 tySNPs, 87 unpublished set of tySNPs for which we previously developed MALDI-TOF MS genotyping assays (Table S2, see *Methods*)** **^31^** **. These 87 tySNPs were a subset of markers identified by the same process except for alignment to the haplotype resolved yak genome.  Their prior selection predated the availability of this novel yak reference assembly.  Thus, their unique alignment and yak genome positions were previously unconfirmed. The positions of the 87 tySNPs, together with the other 523 tySNPs, are shown on the haplotype-resolved yak genome assembly (** **Figure 4** **). Together, all 610 tySNPs, their bins, and their sequence information are suitable for input into SNP assay design software for a variety of genotyping platforms.”**

And we have modified the following within the Discussion

**Page 10, column 2, paragraph 2, line 1 of the first version:**

**Our aim was to identify a set of novel triallelic SNPs. We identified a novel set of 610 tySNPs where each marker has two alleles for NA yak parentage testing, and a third allele for identifying recent cattle introgression.**

As to the reviewer’s suggestion that there may be a better set of 87 markers available within the 610, we agree.  However, the MAF needs to be estimated for each tySNP in NA yak before this can be explored further.  That effort is beyond the scope of this report.  All 610 tySNPs should be considered viable targets for assay development, with anyone being free to use them in full or to identify a suitable subset within them for their specific purpose.

We made the following modification to this sentence in the conclusion:

**Page 13, column 1, paragraph 2, line 6 of the first version:**

**The 610 tySNPs, *their assay design information, *multiplex MALDI-TOF MS assays for the subset of 87 tySNPs, and *all other *associated information are available for world-wide use without restriction.** 

In addition, we have responded point-by-point to your comments below (bold text).  

With respect to requests to make the manuscript briefer, since length is not a concern in this online format, and the editors had previously requested such information, we have left most of the content unchanged.  

Introduction:

 

- The background information is very informative, but perhaps the introduction is a bit too long.
- What is the calculated evolutionary distance (time) between cattle and the yak? 
  - **We modified Page 3 column 2 paragraph 2 to include: In this hypothetical triallelic SNP system, the evolutionary source of all three alleles could be inferred by estimating frequencies in samples of *Bos *species, a genus that diverged 5 million years ago. **
- Both Figure 1A and 1B are not necessary.  Perhaps use Figure 2 as Figure 1B?  
  - **We believe that the Figure 1 panels show different information.  Figure 1A shows how tySNPS would arise, while Figure 1B shows the accurate species distances from the root.  **

Methods:

 

- Sample collection – “purchased or donated from zoological parks in their normal course of caring for animals”. Most zoological parks have ACUC protocols and require “study protocol forms” for samples released – even after opportunistic collections during health examinations. Perhaps the protocols should be confirmed?
  - **The following information was added to Methods: The banteng dna samples (1 ug each) were transferred under a Research Material Agreement that was executed on February 6, 2007 by the legal directors of the San Diego Zoo and the technology transfer office of the USDA, ARS.  The gaur blood samples were collected under IACUC protocols on June 15,1999 at the Omaha Henry Doorly Zoo by the head zoo veterinarian, Dr. Doug Armstrong, during evaluation and preparation for interstate transfer to another zoo.**

 

 

- Can more of the Methods be shortened by using references? Such as details on the identity of the animals used for sequencing?
  - **This journal requires full method descriptions, efforts to shorten with previous submissions have been declined.   **

 

 

- The use of the pedigrees to select a strong representation of the beef cattle breeds is a strong aspect of the project.
  - **We agree.**

 

 

- Correct spacing: “TrimGalore (version 0.5.0)and”.   
  - **Repaired Page 5 column 2, paragraph 2**
- Perhaps a supplementary table could be used to represent the information in the text regarding the number of each breed and the genome accession numbers, thus, shortening the text.
  - **We have left the text as is.**
- “ *the cutoffs for call rate (total geno-types obtained/total genotypes possible) and accuracy (correctgenotypes/total genotypes obtained) were set at 97% and 99%, respectively.”*and “For the purposes of parentage exclusion the cutoff call rate of 97% means that a minimum of 94% of the tySNPs will have a genotype for each of the two animals in a pairwise comparison”These are stringent cutoffs for the data and should produce a very robust panel of SNPs.
  - **We agree.**

 

 

- Used MALDI-TOF mass spectroscopy for genotyping: a preliminary set of 139 bins with 518 tySNPs aligned to the B. taurus UMD3.1 assembly. 48 assays per plex using one SNP per bin – producing 3 multiplexes.
  - **This is correct.**
- Although very thorough – can the section including the “Estimating the probability of identity (PI) and probability of exclusion (PE) with tySNPs”, and “Identifying exclusions in a tri-allelic SNP system” be shortened by referencing?
  - **As described above, F1000Research editors require full method descriptions, efforts to shorten with previous submissions have been declined.**

 

 

- Overall, the bioinformatic approaches appear to be sound and robust for this study.
  - **We agree.**
- Figure 3 is not really needed.
  - **As this work was inspired by input from yak breeders, we wanted to include a figure that shows producers the expected loss of introgressed cattle alleles across generations of yak backcrossing. This figure relates directly to the ability of tySNPs to detect cattle introgression as shown by simulations in Figure 8.**

Results:

 

- Interesting – “ *Of the 610 tySNPs, 87 overlapped an unpublished set of tySNPs for which we previously developed MALDI-TOF MS genotyping assays (Table S2, see Methods)31*.” Thus, this panel was already developed and in use, but then verified by phased yak genome. Therefore – how were these original 87 tySNPs identified and designed?
  - **Addressed in the revised manuscript in “Amendments from Version 1”. **

 

 

- If starting from scratch with 610 tySNP candidates and then designing mass array multiplexes – 441 bins with 48 SNPs per multiplex = 10 assays minimum.
  - **Depending upon the total number of assays required by an interested party, and the assay platform/chemistry, any subset of the proposed bins could be chosen for an assay panel.  We have demonstrated with a MALDI-TOF MS platform that 3 multiplexes with 87 total assays will provide enough information to reasonably accomplish animal identification, parentage determination, and detect recent hybridization.** 

 

 

- Thus – it does not seem like the authors used the presented information to produce the 87 tySNP panel. After the fact – they are validating the panel but not suggesting any improvements from the data produced by the methods performed in the manuscript. For example, at least 5 or so of the 87 tySNPs appear to be very physically close in Figure 4 – and hence should not be in the panel.
  - **As addressed in the revised manuscript in “Amendments from Version 1”, our aim was not to validate an elite panel of 87 tySNPs, but to use the population genotypes to estimate their MAF in NA yak.  Since all tySNPs were selected based on being heterozygous in only two yaks (1 NA and 1 Chinese), we had initial concerns that the MAF for most of the SNPs would be too low to be useful.  Using data from the 87 unmapped tySNPs and 170 yak we were pleasantly surprised there was a useful distribution of MAFs.  We document this in Figure 5A and can reasonably infer a similar distribution among all 610 tySNPs.**

 

 

- The *in silico* analysis for the 610 SNP allele frequencies confirmed the allelic frequencies for the 87 SNPs that were genotyped in the 170 yaks. However, could a better set of tySNPs have been selected from this data?
  - **Any new panel design starting with these 610 tySNPs would likely end with a different marker composition.  However, any randomly selected set of 87 tySNPs distributed among the 441 bins are expected to produce similar results in NA yaks. **
  - **Moreover, creating specific combinations of markers into multiplex assays (up to 48 at a time) is a tremendous technical challenge.  This challenge is increased by having three SNP alleles to assay instead of just two.  In addition, flanking polymorphisms near some of the tySNPs reduce the utility of some of the 610 identified tySNPs depending on the genotyping platform and assay requirements.  All of these challenges were overcome here as part of a solution to develop as powerful of assay as possible with cost awareness.**

 

 

- All the exclusion and introgression calculations are based on the 87 SNPs.
  - **Since we had data on 87 SNPs, that number was used as the lower limit.  In order to demonstrate the benefit of more markers, we simulated tests with 1.5, 2, and 4-fold increases in marker count.  The balance between cost and the benefit of more markers is difficult to strike.  However, we wanted to demonstrate what could be expected with these 87 tySNPs since this is the data we had.  **

 

 

- No animals with documented, known hybridization were genotyped for validation.
  - **This assertion is incorrect.  We used the F1 cross between a Highland bull and a yak dam for in-silico genotyping of the full panel of 610 markers (Table 1 last row).   This hybrid female (“Esperanza”) had a 0.5049 cattle allele fraction.  The genotypes are provided in Table S3.  **

Discussion:

 

- *“Our aim was to identify minimal set of novel triallelic SNPs to accomplish multiple diverse genetic tests in NA yaks.”*The authors do define a robust set of triallelic SNPs – however – most robust or minimal set has NOT been identified because this paper confirms an *a priori* set of 87 SNPs that were selected using perhaps a different process as described here. The authors need to confirm the same process was used to select the 87 SNPs – short of using the phased yak assembly for confirmation and positioning, which they indicate.
  - **We confirm.**
- This manuscript does indicate the 87 SNPs developed for yak testing are robust and have good exclusion rates, but it does not select the best SNPs from the 610 to produce the panel. No modifications are suggested for the panel – including for SNPs now shown to be in close proximity or the 6 tySNPs with *B. taurus* – associated allele frequencies. However, the authors do suggest that better SNPs could be selected in more yak WGS data was available. Another way to produce this data would be to make additional multiplexes from the remaining 610 SNPs and genotype the same 170 yaks. If 87 SNPs had already been produced – likely – additional data on tySNPs not selected for the panel is likely available. 
  - **The success of SNP assay design is dependent on many factors including chemistry, platform, technology, reagents, sample quality, and operator experience, to name a few.  We used *all* the data from *all* the tySNPs that met the selection criteria and converted to a reliable assay (>97% call rate and >99% accuracy).  Figure 5A shows that some of these 87 tySNPs had low MAFs which is less than ideal for the tasks at hand but were still included.  As mentioned above, any new panel design starting with these 610 tySNPs would likely end with a different marker composition.  However, any random set of 87 tySNPs distributed among the 441 bins are expected to produce similar results in NA yaks.  **

 

 

- In the end, the NA yak are not significantly influenced with *B. taurus* introgression, thus, a standard set of SNPs would be likely sufficient, although introgression could not be monitored.
  - **As the reviewer indicates, introgression cannot be monitored with a standard set of bi-allelic SNPs.  This was the motivation to identify tySNPs that can detect recent introgression among NA yak without the need for a second, independent set of markers for that task. **
